# Identification of Drivers from Cancer Genome Diversity in Hepatocellular Carcinoma

**DOI:** 10.3390/ijms150611142

**Published:** 2014-06-20

**Authors:** Atsushi Takai, Hien T. Dang, Xin W. Wang

**Affiliations:** Laboratory of Human Carcinogenesis, Center for Cancer Research, National Cancer Institute, National Institutes of Health, Bethesda, MD 20892, USA; E-Mails: atsushi.takai@nih.gov (A.T.); hien.dang@nih.gov (H.T.D.)

**Keywords:** hepatocellular carcinoma, tumor heterogeneity, cancer driver gene

## Abstract

Hepatocellular carcinoma (HCC) is one of the most common cancers with a dismal outcome. The complicated molecular pathogenesis of HCC caused by tumor heterogeneity makes it difficult to identify druggable targets useful for treating HCC patients. One approach that has a potential for the improvement of patient prognosis is the identification of cancer driver genes that play a critical role in the development of HCC. Recent technological advances of high-throughput methods, such as gene expression profiles, DNA copy number alterations and somatic mutations, have expanded our understanding of the comprehensive genetic profiles of HCC. Integrative analysis of these omics profiles enables us to classify the molecular subgroups of HCC patients. As each subgroup classified according to genetic profiles has different clinical features, such as recurrence rate and prognosis, the tumor subclassification tools are useful in clinical practice. Furthermore, a global genetic analysis, including genome-wide RNAi functional screening, makes it possible to identify cancer vulnerable genes. Identification of common cancer driver genes in HCC leads to the development of an effective molecular target therapy.

## 1. The Landscape of the Cancer Genome

Many studies over the past century have revealed that cancer is a genetic disease. Most cancers are thought to develop through the acquisition of genetic alterations and the selection of neoplastic clones in a preferred tumor microenvironment [1,2,3]. Over time, a few unique clones will acquire the ability to proliferate autonomously, invade tissues and metastasize to distant organs. Recent technological advances in DNA sequencing of cancer tissues revealed that the cancer genome is more heterogeneous across tumor types than we have imagined. Tumor heterogeneity can be seen amongst different patients with the same tumor type, between different tumor nodules from the same patient and within the same tumor mass. This new knowledge affects the classification of cancer subtypes. For example, the conventional classification of breast cancers is based on histopathological grade and tumor type, immunohistochemical analysis of hormone receptors and overexpression of human epidermal growth factor receptor 2. These categories have been extended by molecular profiling analyses, which classify breast cancers along with unique biological and prognostic characteristics [4]. Moreover, comprehensive genome sequencing studies, such as The Cancer Genome Atlas (TCGA), have identified numerous mutations in various types of cancers. As expected, the number and the pattern of mutations vary greatly amongst different patients and tumor types [5,6,7,8,9,10,11,12,13,14,15]. Taken together, tumor heterogeneity is a significant contributory factor that makes it difficult to treat cancer patients with a “one size fits all” approach. Then, how can we confront this formidable challenge? One way to directly address tumor heterogeneity is to develop molecularly targeted therapies by identifying tumor vulnerabilities for each tumor subtype with a relatively homogenous tumor biology. Genetic alterations detected in tumors are generally classified into two groups: driver mutations or passenger mutations. A driver mutation is evolutionarily selected during tumorigenesis and confers a growth advantage on cancer cells. Passenger mutations are acquired and do not contribute to cancer development. A recent study reported that at least 1.6% of the protein-coding genes in the human genome showed recurrent somatic mutations that may contribute to cancer development [16]; however, most mutations were non-functional passengers. Therefore, it is important to identify cancer driver genes from the pile of genetic information for molecular-targeted therapy from non-functional passengers, which may have no effect on cancer treatment. This review summarizes the current knowledge of tumor heterogeneity and cancer driver genes, specifically in hepatocellular carcinoma (HCC).

## 2. Heterogeneity of Genetic Alterations in Hepatocellular Carcinoma (HCC)

The development of HCC is closely associated with multiple risk factors, such as chronic infection of hepatitis B virus (HBV) [17,18] and hepatitis C virus (HCV) [19,20], alcohol consumption [21,22,23], aflatoxin B1 exposure [24,25,26,27,28] and obesity [29]. In addition to these common etiological factors, other factors are known to contribute to hepatocarcinogenesis with a low frequency, including non-alcoholic fatty liver disorders, diabetes, hemochromatosis and long-term oral contraceptive use [30,31,32,33,34]. These etiologic factors impact the molecular characteristics of HCC. While tumor heterogeneity is found amongst HCC patients, the tumor itself consists of cancer cells with various genetic profiles (intra-tumor heterogeneity). Similar to other solid tumors, a number of genetic alterations accumulate during the development of HCC. Genetic alterations accumulate slowly in a limited number of genes and chromosomal loci during the early preneoplastic stage and accelerate throughout dysplasia and into HCC. Allelic deletions have been identified in 30%–50% of liver tissues with chronic hepatitis or cirrhosis, 70%–80% of dysplastic lesions and all of the HCC nodules [35,36,37,38]. The fact that cells within the cirrhotic lesions and adjacent HCC nodules have acquired genetic alterations is not indicative of cell populations harboring driver alterations that may evolve into a malignant phenotype, since many are passengers [30]. According to previous studies, only a small number of pathways or genes, such as p53, Wnt/β-catenin and *RB* [39], are known to be responsible for hepatocarcinogenesis. Moreover, analyses using partial lesions of tumor tissues collected from individual patients provide only a snapshot of the complicated profiles that the tumor possesses. The molecular pathogenesis of HCC is so complicated, that the identification of critical molecular pathways of carcinogenesis is challenging. However, recent technological development of high-throughput analysis tools enables us to view the comprehensive genetic profiles of HCC.

## 3. Gene Expression Profiles in HCC

The advent of DNA microarray and RNA sequencing technologies has made a notable impact on the field of genetics. Microarray gene expression technology has allowed the analysis of thousands of genes, covering the whole genome in different tissue types [40]. One of the early studies using microarray technology demonstrates that global gene expression patterns within HCC tumors were completely different. This difference was also evident amongst multiple nodules from the same patient [41]. Subsequently, numerous studies for gene expression profiles within HCC have been presented during the last decade [42]. While there are differences amongst HCC, hierarchical clustering analysis of tumor-specific genes can classify different HCC subtypes. This molecular classification will provide a benefit to the clinic, as well as unravelling the complex pathogenesis of HCC, since these subgroups differ according to etiological factor [43,44,45], the clinical stage [46,47], recurrence rate [48] and prognosis [49]. Thus, hierarchical clustering analysis proves to be a powerful tool for discovering different HCC subtypes associated with clinical outcome.

There are two main types of subgroup discovery approaches, *i.e.*, unsupervised and supervised. Unsupervised clustering strategies incorporate the expression profiles of the whole transcriptome, presenting a comprehensive landscape of the molecular features of every subclass, and can potentially discover novel cancer driver genes. For example, Boyault *et al.* [50] performed global transcriptome analyses on 57 HCC cases and identified six robust subgroups (G1–G6) associated with clinical and genetic features. Several types of genetic alterations were detected, which was closely associated with the subgroups. Interestingly, the authors were able to show that G2–G3 subgroups were associated with *TP53* mutations, whereas G5–G6 subgroups were associated with *CTNNB1* mutations. Moreover, patients within the G1–G3 subgroups were HBV-positive and showed evidence of high chromosomal instability when compared with the G4–G6 subgroups. Notably, within the G1–G3 subgroup, patients in the G1 alone had a lower HBV DNA copy number compared to all subgroups. In an independent study, Lee *et al.* analyzed the expression profiles of 91 HCC cases and identified two subgroups with a significant difference in survival rate [49]. They identified 406 unique genes that are closely associated with survival and important for cell cycle regulation, cell proliferation and apoptosis. This suggests that there may be cancer driver candidates amongst the 406 tumor-specific genes, which may functionally contribute to tumor progression and survival. Accordingly, a meta-analysis of gene expression data from eight independent cohorts by Hoshida *et al.* [51] identified three robust and stable HCC subclasses (S1–S3) that are correlated with clinical features, such as tumor size, tumor differentiation and serum α-fetoprotein (AFP) level. Furthermore, each subclass had unique molecular characteristics, such as activation of the Wnt/β-catenin pathway in S1, prominent activation of MYC and AKT in S2 and hepatocyte differentiation in S3.

On the other hand, supervised clustering strategies are useful for the determination of subgroups based on particular biological or clinical features, as well as identifying potent cancer drivers from the specific candidate gene lists. Ye *et al.* [47] analyzed the gene expression profiles of HCC samples with or without intra-hepatic metastasis and discovered a 153-gene signature that can classify metastatic HCC and predict tumor recurrence after tumor resection. Among those, osteopontin was the lead candidate gene that was functionally important in HCC metastasis. In an independent study by Budhu *et al.* [52], the authors identified unique genetic profiles associated with HCC metastasis by comparing gene expression profiles of non-cancerous tissues of HCC patients with venous or extrahepatic metastasis and those without metastasis. The unique signature was found in non-cancerous tissues that were mostly important for inflammation/immune response. Moreover, a refined 17-gene signature was identified as a predictor of HCC metastasis and tumor recurrence in an independent cohort [52]. Together, these studies indicate that gene expression analysis can classify patients into subgroups and help identify candidate cancer driver genes by incorporating clinical information with genetic data. This type of data analysis can also be used to discover the different cellular origins of the tumor, which has been proposed to contribute to different oncogenic signaling pathways during tumorigenesis and patient survival [53]. Lee *et al.* [54] integrated gene expression data from rat fetal hepatoblasts and human HCC cells. Interestingly, the authors demonstrated that patients with HCC share the same gene expression pattern with rat fetal hepatoblasts (HB subtype) and had a poor prognosis compared to other HCC patients (HC subtype). Their data revealed that 907 genes were differentially expressed between HB and HC subtypes. Interestingly, Lee *et al.* showed that these genes included markers of hepatic progenitor cells, such as KRT7 and KRT19. Their results suggest that the HB subtype may have arisen from hepatic progenitor cells, which possess the capacity to differentiate into mature hepatocytes or cholangiocytes. In contrast, the HC subtype was derived from differentiated hepatocytes.

## 4. Copy Number Alterations in HCC

The phrase “genetic alterations” comprises wide-ranging implications from amplifications or deletions of chromosomal arms to point mutations of each gene. Chromosomal aberrations are commonly observed in tumors, and the selection of clones with genetic traits that have an advantage for tumor development have been demonstrated to contribute to tumor progression [55,56]. The comparative genomic hybridization method (CGH) has shown to be able to survey chromosomal aberrations by detecting somatic DNA copy number changes in cancer genomes. According to Moinzadeh *et al.* [57], who have reviewed 31 CGH from 785 HCC cases, the chromosome arms, 1q, 6p, 8q, 17q and 20q, were frequently amplified, whereas 4q, 8p, 13q, 16q and 17p were commonly deleted. Interestingly, studies have demonstrated a significant correlation between several chromosomal defects and tumor grade, as well as HBV etiology [57]. However, the traditional CGH cannot detect amplifications or deletions less than 2 or 10 Mb, respectively, thus failing to identify smaller chromosomal changes [58,59]. These limitations have been overcome by technological development, such as array-based CGH (array CGH), which enables high-throughput and -resolution screening of genome-wide DNA copy number changes [60] and exon sequencing. The microarray platform used for array CGH makes it possible to scan a large number of DNA sequences from the whole genome and produces high-resolution data. Recently, several studies reported chromosomal alterations in HCC using array CGH [61,62,63,64]. A meta-analysis of 159 HCC array CGHs was able to discover significant gains in 5p15.33 and 9q34.2–34.3 and losses in 6q, 9p and 14q in addition to the regions that were previously identified by conventional CGH analyses [65]. Furthermore, pathway analysis indicated that the genes located on the altered chromosomal arms were enriched in 31 canonical pathways. While most of the pathways are related to antiviral immunity, several cancer-related genes, such as *TP53*, *RB1* and *MYC*, were mapped to the pathways [65]. Although technological development improved the ability to map altered chromosomal regions, numerous genes are included in gain or loss regions, even in a small region, which makes it challenging to identify cancer driver genes for the crowd of candidates. More importantly, studies have demonstrated that gene expression profiles are subject to chromosomal bias [66,67,68,69]. Moreover, genes found within regions of chromosomal aberrations whose gene expression levels are altered are more likely to be oncogenes or tumor suppressor genes. In order to identify candidate genes that directly affect HCC development, integrative analysis of gene copy number with gene expression data should be performed. A study by Patil *et al.* [62] nicely demonstrated the correlation between DNA copy numbers and gene expression pattern at the 8q region, which was frequently amplified in 49 HCC samples. Amongst the 48 genes located on the 8q arm whose expression levels were also elevated, Jab1 (also known as COP9 signalosome subunit 5) showed significant correlation. Notably, Jab1 was functionally important for the development of HCC and has been reported to be associated with the wound response signature in breast cancer [70]. Woo *et al.* [71] integrated whole genome copy number profiles of 15 HCC cases with gene expression profiles of 139 HCC cases to identify potential cancer driver genes. By analyzing genes that have a correlation between expression levels and copy number changes, especially in the regions with common recurrences, the authors discovered 50 potential driver genes that are associated with HCC prognosis. Pathway analyses revealed that these genes were linked to signaling pathways, such as mTOR, AMPK and EGFR. In a study by Roessler *et al.* [72], the authors integrated high-resolution array CGH data and gene expression profiles of 256 HCC cases to assess the correlation between somatic copy number alterations and gene expression patterns across the whole genome to identify potential cancer driver genes. Integrative analysis was able to identify 10 genes that were associated with poor survival. The authors also show that six of the 10 genes were located at 8p, w hich were deleted in patients with poor prognosis. Furthermore, Roessler *et al.* identified three novel tumor suppressor genes (*PROSC*, *SH2D4* and *SORBS3*) and confirmed the tumor suppressive effect of *DLC1* using functional assays.

## 5. Somatic Mutations in HCC

As in many solid tumors, a great number of nucleotide alterations were reported in HCC. So far, several genes, such as *TP53*, *CTNNB1* and *AXIN1*, are frequently mutated and have been demonstrated to contribute to tumor development. Although *TP53* is the most frequently altered gene in HCC [73,74,75,76], its mutation rate is different between geographical regions. While a high frequency of mutations has been reported in patients from East Asia and Africa, it is relatively lower in other countries, including the West [77]. In particular, a specific mutation in codon 249 resulting in a G to T transversion was common in areas of aflatoxin B1 dietary exposure [26,27]. In contrast, HCC patients without aflatoxin B1 exposure had a lower prevalence of *TP53* mutations, except in hemochromatosis-associated HCC [76,78,79]. *TP53* mutations of HCC patients in western countries have been reported to be correlated with tumor grade, clinical stage and prognosis, suggesting that these mutations are not causative of carcinogenesis, but features acquired during tumor progression [80,81,82].

Currently, the dysregulation of Wnt/β-catenin signaling has been observed in a subset of HCC. Nucleotide alterations of *CTNNB1* have been found in 13%–44% of HCC cases [73,83,84,85,86]. In addition, other genes within the Wnt/β-catenin signaling pathway, such as *AXIN1* and *AXIN2*, were also reported to be frequently mutated [73,84,87,88]. These mutations activate the Wnt/β-catenin signaling cascade by stabilizing the elevated expression of β-catenin. Accordingly, Hsu *et al.* [83] reported that *CTNNB1* mutations were associated with non-HBV-related HCC, lower stage and good prognosis, suggesting that the mechanisms of β-catenin mutations are different between HBV-related and non-HBV-related HCCs and that β-catenin mutation may be a favorable prognostic marker.

Highly-frequency somatic mutations were also identified at the promoter region of specific genes. Nault *et al.* [89] recently reported recurrent mutations of the telomerase reverse-transcriptase (*TERT*) promoter in 59% of HCC cases, 25% of cirrhotic preneoplastic macronodules and 44% of hepatocellular adenomas with malignant transformation. The authors found that *TERT* promoter mutations in HCC were significantly associated with *CTNNB1*-activating mutations.

Although previous studies established the direct effect of the mutations in these genes in cancer development, more than half of the HCC cases are not associated with driver mutations. Recent innovation in sequencing technology has expanded our knowledge of the global genomic landscape of multiple diseases, including malignancy [90,91]. The comprehensive genetic analyses using second-generation DNA sequencing methods increased the ability to detect somatic cancer genomic alterations with high resolution. During the past half-decade, a number of somatic mutations have been detected in human cancers by genome-wide sequence studies. In HCC, several studies of whole-genome and exome analyses have been performed to provide a comprehensive landscape of genetic alterations. According to the 27 cases in the Japanese cohort [92], 110 cases in the Chinese cohort [93] and 125 cases in the French cohort [94], *TP53* was the most commonly mutated gene with a high frequency (21%–52%). On the other hand, *CTNNB1* mutation frequency in the Japanese cohort (11%) and French cohort (33%) were significantly higher than the Chinese cohort, which consists of HBV-related samples. Consistent with these findings, alterations of the *CTNNB1* gene were barely detected in HBV-related HCC samples compared to those without HBV infection in the French cohort [94]. Interestingly, *CTNNB1* mutations were mutually exclusive from the *TP53* mutations [94]. In contrast, *AXIN1* and *APC* mutations, which were detected in 15.2% and 1.6% of cases in the French cohort, respectively, were not associated with specific etiological factors and were not exclusive from *TP53* alterations [94]. Notably, current studies have identified a high frequency of somatic mutations in the *ARID1A* gene in all three cohorts (11% of Japanese cohort, 13% of Chinese cohort and 17% of French cohort). ARID1A is one of the components of the SWItch/Sucrose NonFermentable (SWI/SNF) chromatin remodeling complexes, which is responsible for remodeling nucleosomes and modulating transcription efficiency [95,96]. *ARID1A* belongs to the *Brahma-related gene 1* (*BRG1*)-associated factor, which is a core subunit of the SWI/SNF complexes, and was recently found to be mutated in several tumor types, including ovarian, bladder and gastric cancers [97,98,99,100,101]. Furthermore, somatic mutations located within the functional domains have been identified to be predictive of an inactivate ARID1A, confirming earlier reports of the low levels of *ARID1A* gene expression reported in other cancers [94]. Moreover, studies have confirmed that *ARID1A* gene knockdown functionally promoted the proliferation, migration and invasion of HCC cells [93], suggesting that *ARID1A* is a tumor suppressor. Another chromatin remodeling gene, *ARID2*, whose protein product belongs to the polybromo BRG1-associated factor of SWI/SNF complexes, was also mutated at a high rate in two cohorts [94,102]. More than 24% of HCC cases contained a mutation in genes associated with chromatin remodeling in the French cohort [94]. The disruption of the chromatin signaling pathway may contribute to HCC development through the modulation of the epigenetic state; therefore, chromatin remodeling-related genes could be candidates for molecular targeted therapy for the treatment of HCC.

Table 1 summarizes candidate driver genes of HCC based on mutations profiles from deep sequencing, the COSMIC database (v.69, http://cancer.sanger.ac.uk/cancergenome/projects/cosmic/), gene expression profiles and chromosomal aberrations.

**Table 1:** Summary of current cancer driver candidates in hepatocellular carcinoma (HCC).

Cell Function	Gene Name	Frequency of Mutations		Gene Expression Dysregulation	Chromosomal Alterations the Gene Is Located
		Deep Sequencing *	COSMIC Database		
Cell cycle	*TP53*	48%, 27%, 21%	31%	Decreased **	17p loss ^†^
	*IRF2*	0%, 0%, 5%	1%	Unreported	4q loss ^††^
	*CDKN2A*	0%, 0%, 7%,	10%	Decreased ^‡^	9p loss ^‡‡^
Cell proliferation	*CTNNB1*	11%, 0%, 33%	19%	Increased ^¶^	Unreported
	*AXIN1*	0%, 0%, 15%	13%	Decreased ^¶¶^	16p loss ^§^
	*KRAS*	0%, 0%, 1.6%	2%	Unreported	Unreported
	*PIK3CA*	7%, 0%, 1.6%	6%	Unreported	Unreported
	*ERRFI1*	7%, 0%, 0%	2%	Unreported	1p loss ^§§^
Chromatin remodeling	*ARID1A*	11%, 13%, 17%	14%	Unreported	Unreported
	*ARID2*	7%, 4%, 6%	9%	Unreported	Unreported

References: * [92,93,94]; ** [103]; ^†^ [62,65,104,105,106,107,108,109]; ^††^ [62,65,94,104,105,106,107,108,109,110,111,112]; ^‡^ [113]; ^‡‡^ [65,94]; ^¶^ [114,115,116]; ^¶¶^ [117]; ^§^ [94]; ^§§^ [104,105,106,108,118].

## 6. The Screening of Cancer Drivers

The elucidation of the comprehensive genetic profiles of cancer cells has led to the identification of potential cancer driver genes. However, passenger genes that are coamplified with driver genes have also been identified as candidates for drivers. Therefore, a genome-wide functional screening strategy may prove useful for identifying genes that directly have an effect on the fate of tumors. Several studies have utilized mouse models of HCC to search for driver genes. For example, Sawey *et al.* [119] developed a unique screening method that assesses the oncogenic potential of candidate driver genes from amplicons found in human HCC. The authors selected 29 recurrent focal amplicons from the array CGH analysis of 89 human HCC samples and 12 HCC cell lines. From the set of amplified genes within the recurrent focal amplicons, a retroviral expression library of 124 full-length cDNAs was constructed and introduced into immortalized murine embryonic hepatoblasts lacking p53 and over-expressing MYC to evaluate their tumorigenic effect. As a result, 18 genes were validated as tumor-promoting genes, which included well-established oncogenes previously implicated in HCC, such as CCND1 and MET [120,121]. In addition, the authors demonstrated that FGF19, which is located at a nearby site of CCND1, is oncogenic in patients with 11q13.3 amplification. In another study by Zender *et al.* [122], the authors utilized an RNA interference (RNAi) strategy for screening genes responsible for HCC development in mice. They selected 631 shRNAs targeting 362 genes, which were identified as candidates of tumor suppressor genes from array CGH analysis of human HCC samples. The shRNA pools were introduced into murine hepatoblasts lacking p53 and over-expressing MYC. The infected cells were injected into the liver of *p53*^−/−^; *Myc* mice, and their tumor genetic effects were evaluated. Using this approach, they have identified and validated 13 tumor suppressor genes, 12 of which had not been previously reported. In a study by Shramek *et al.* [123], the authors selected 1762 lentiviral shRNAs, which targets 347 candidate driver genes, to inject into E9.5 embryos of TGFβ-receptor-II conditional knockout mice, susceptible to the development of squamous cell carcinomas (SCCs), *in utero*. These transduced mosaic mice developed SCCs, and sequencing analysis revealed that independent shRNAs specific for eight genes are highly enriched in multiple tumors. In a study by Bard-Chapeau *et al.* [124], the authors performed a unique method called “transposon mutagenesis screen”. They utilized mutagenic transposons carrying a strong promoter for activating proto-oncogenes and transcriptional stop cassettes for inactivating tumor suppressor genes for identifying cancer driver genes in HCC. The mutagenic transposons were mobilized in the livers of transgenic mice with constitutive expression of HBV surface antigen in their livers. This screen identified 21 candidate genes of early stage HCC and 2860 candidates for late stage, respectively. These *in vivo* forward-genetic screens can be utilized for genome-wide identification of cancer driver genes from activating genetic alterations identified by human cancer genome profiling.

Recently, new high-throughput technology that combines the RNAi strategy with microarray gene expression analysis has been developed as a tool for the exploration of cancer driver genes. The complex pool of shRNA libraries labeled with unique barcodes are hybridized with specific probes on the microarray platform. Pioneering studies of this genome-wide RNAi screening were performed using colon and breast cancer cells. In a study by Schlabach *et al.* [125], the authors used a highly complex pool of 8203 shRNAs to target 2924 genes in normal and colon cancer cells to evaluate the relative abundance of each shRNA. Accordingly, Schlabach and colleagues identified 23 anti-proliferative shRNAs that were common in four different colon cancer cell lines. In an independent study, Silva *et al.* [126] screened 6000–20,000 shRNAs using five breast cancer cell lines and identified common lethal shRNAs that targets well-established cell cycle regulatory networks. Furthermore, RNAi screening is also useful for the identification of genes whose loss of function constitutes synthetic lethality with a particular oncogenic state. For example, Luo *et al.* [127] screened the shRNA library for genes whose inhibition constitutes synthetic lethality with the mutated *KRAS* gene in colon cancer cells. Luo and colleagues transfected 74,905 shRNAs targeting 32,293 transcripts in colon cancer cell lines with or without *KRAS* mutation (Ras Mut and Ras WT). The authors analyzed the change in abundance of each shRNA over time by microarray hybridization to identify shRNAs that were depleted due to their antiproliferative effect to generate a lethality gene signature. This lethality signature consists of 379 candidate shRNAs that were selectively depleted in Ras Mut cells, but not in WT cells. A total of 83 shRNAs were functionally validated to have a synthetic lethal effect with Ras Mut cells. A number of genes with mitotic functions, such as polo-like kinase 1, subunits of APC/C complex and cyclin A2, were considered as potential Ras synthetic lethal genes. Their results demonstrated how a genome-wide RNAi screening may be useful for identifying potential therapeutic markers in tumor with particular oncogenic alterations. This screening method is also applicable to other malignant diseases, including HCC.

## 7. Future Directions

**Figure 1:**
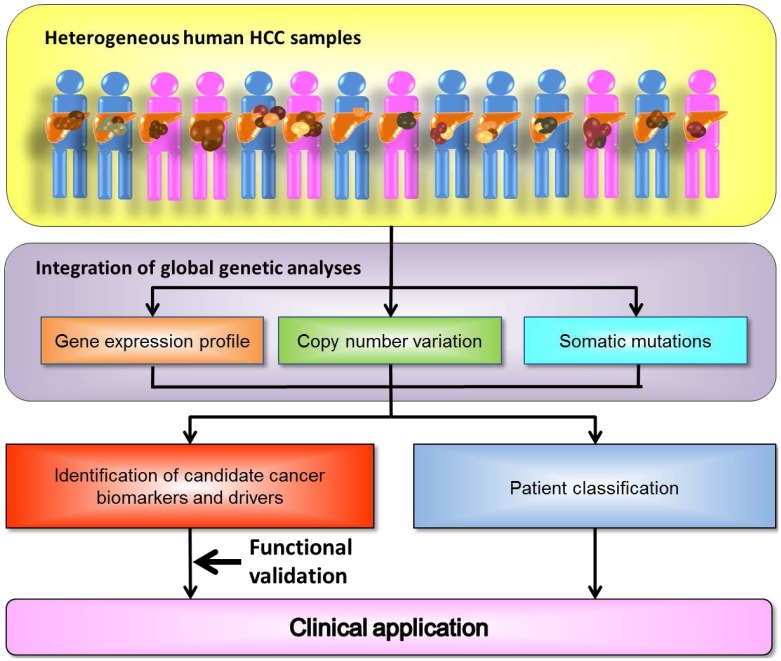
Strategies for the identification of cancer driver genes or alterations. The integration of comprehensive genetic analyses will be useful for discovering a target molecule applicable to HCC treatment.

The molecular pathogenesis of HCC is complex, making it difficult to elucidate the underlying mechanism of tumorigenesis. Tumor heterogeneity amongst patients is a major contributor to the complex HCC genome. The comprehensive analysis of the genetic landscape using tumor samples of HCC patients with various etiologies provides us with much information about the molecular features that each individual HCC tumor possesses. By identifying the cancer drivers and elucidating the genetic landscape of HCC, we will be able to understand how tumor heterogeneity may affect, and possibly confound, targeted therapeutic interventions, such as *HER2* amplification in breast cancer cases [128]. The integration of data from different levels of global analyses, as well as RNAi functional screening will be useful for identifying common molecular events required for tumor progression (Figure 1). On the other hand, whole human genome sequence technology enables us to identify single nucleotide polymorphisms (SNPs) that each individual patient possesses. More refined precision medicine will be offered if the pathogenic role of each SNP is revealed. Precision medicine based on global genetic analysis will become more important to overcome the heterogeneity of HCC.

While some genetic profiles or signaling pathways may prove to be potential targets for clinical application, the molecular characteristics of HCC vary widely among patients. The existence of intra-tumor heterogeneity can be subjected to recurrence even after the eradication of most cancer cells. Each cancer cell inside the tumor has acquired deferent molecular features during tumorigenesis. A new paradigm of how cancer develops has implicated the existence of cancer stem cells. These cells are resistant to chemotherapy and may prevent patients from achieving complete remission. The establishment of the molecular target therapy that selectively kills cancer stem cells will be required to solve the problem of intra-tumor heterogeneity. Although the knowledge about the mechanism of how cancer stem cells are maintained is still limited, a better understanding about the behavior of cancer stem cells will lead to the development of revolutionary therapies that selectively eradicate malignant cells responsible for cancer initiation.

The heterogeneity of HCC makes it difficult to clarify the mechanism of cancer development and to develop effective therapeutics. However, an integrative functional genomics approach will contribute to the discovery of potential molecular features critical for HCC development. These studies will provide us with better treatment strategies that may be effective to treat all HCC patients.
